# The anion exchanger slc26a3 regulates colonic mucus expansion during steady state and in response to prostaglandin E_2_, while Cftr regulates de novo mucus release in response to carbamylcholine

**DOI:** 10.1007/s00424-024-02975-4

**Published:** 2024-06-03

**Authors:** Penny L. Ljungholm, Anna Ermund, Molly M. Söderlund Garsveden, Victor L. Pettersson, Jenny K. Gustafsson

**Affiliations:** 1 Department of Physiology, University of Gothenburg, Medicinaregatan 11, Box 432, 405 30 Gothenburg, Sweden; 2 Department of Medical Chemistry and Cell Biology, University of Gothenburg, Gothenburg, Sweden

**Keywords:** Colon, Ileum, slc26a3, Anion transport, Cftr, Cl^−^/HCO_3_^−^ exchange, Mucus secretion, Mucus expansion

## Abstract

The intestinal epithelium is covered by mucus that protects the tissue from the luminal content. Studies have shown that anion secretion via the cystic fibrosis conductance regulator (Cftr) regulates mucus formation in the small intestine. However, mechanisms regulating mucus formation in the colon are less understood. The aim of this study was to explore the role of anion transport in the regulation of mucus formation during steady state and in response to carbamylcholine (CCh) and prostaglandin E_2_ (PGE_2_). The broad-spectrum anion transport inhibitor 4,4′-diisothiocyanatostilbene-2,2′-disulfonate (DIDS), CftrdF508 (CF) mice, and the slc26a3 inhibitor SLC26A3-IN-2 were used to inhibit anion transport. In the distal colon, steady-state mucus expansion was reduced by SLC26A3-IN-2 and normal in CF mice. PGE_2_ stimulated mucus expansion without de novo mucus release in wild type (WT) and CF colon via slc26a3 sensitive mechanisms, while CCh induced de novo mucus secretion in WT but not in CF colon. However, when added simultaneously, CCh and PGE_2_ stimulated de novo mucus secretion in the CF colon via DIDS-sensitive pathways. A similar response was observed in CF ileum that responded to CCh and PGE_2_ with DIDS-sensitive de novo mucus secretion. In conclusion, this study suggests that slc26a3 regulates colonic mucus expansion, while Cftr regulates CCh-induced de novo mucus secretion from ileal and distal colon crypts. Furthermore, these findings demonstrate that in the absence of a functional Cftr channel, parallel stimulation with CCh and PGE_2_ activates additional anion transport processes that help release mucus from intestinal goblet cells.

## Introduction

The intestinal epithelium is a dynamic structure capable of alternating between absorption and secretion to fine-tune ion and water homeostasis and to maintain a protective barrier against the large amount of microorganisms that reside in our gastrointestinal tract [16, 23, 24]. Coordinated secretion of ions, fluid, and mucus each contribute to maintaining the intestinal barrier. Anion secretion provides the driving force for fluid secretion and regulates the ionic milieu required for normal mucus layer formation [1, 15, 30]. Building of the intestinal mucus barrier is a multi-step process starting with exocytosis of the mucin granules, followed by expansion and processing of the secreted mucins to form the mucus barrier [12, 18, 49]. In the small intestine, the mucus forms a loose and permeable structure that acts as a diffusion barrier for antimicrobial proteins and peptides and a habitat for the microbiota [7, 13, 18, 29]. The loose structure of the small intestinal mucus makes it easily transported in the distal direction which combined with the secretion of antimicrobial proteins and peptides keeps the amounts of bacteria lower in the small intestine as compared to the colon [36]. In the mid and distal colon of mice, the mucus forms a dense adherent barrier that separates the vast majority of the commensal microbiota from accessing the underlying epithelium [5, 24, 25]. Thus, the properties of the mucus layers differ between the small intestine and colon despite the fact that the main structural component of the mucus layer, mucin 2 (Muc2), is shared by the two organs [25]. The physiological importance of secreting mucus with the correct properties is exemplified in the disease cystic fibrosis where the loss of a functional cystic fibrosis conductance regulator (CFTR) that transports chloride and bicarbonate results in mucus plugging in CFTR-expressing organs such as the airways, small intestine, pancreas, and reproductive tract, which in turn leads to inflammation, and subsequent organ damage [10]. Studies in the small intestine and airways of mice and pigs have shown that loss of Cftr-mediated anion transport, in particular bicarbonate, renders the small intestinal and airway mucus into a dense and adherent layer that acts as a binding point, and nutrient source for bacteria, resulting in bacterial overgrowth [8, 15, 32, 49]. In the colon, where the mucus normally is dense and adherent, loss of CFTR-mediated transport causes less severe pathology as compared to the small intestine, indicating that additional transport processes are involved in regulating mucus layer formation in the colon [11]. Studies have shown that Tmem16A (Anoctamin 1), a Ca^2+^ regulated anion channel, is involved in regulating baseline mucus release in the mouse distal colon; however, whether other anion transporters such as the anion exchanger slc26a3 (down-regulated in adenoma (DRA)) is involved in regulating other aspects of mucus layer formation such as expansion of the mucus following exocytosis or regulates de novo mucus secretion in response to secretagogues is not known [4]. In the present study, we explored the role of anion transport in the regulation of mucus layer formation during steady state and in response to the muscarinic receptor agonist carbamylcholine (CCh) and prostaglandin E_2_ (PGE_2_). To assess the role of anion transport in regulation of mucus layer formation, we measured mucus growth over time, and mucus adhesion in ileal and colonic intestinal explants ex vivo. We have previously shown that in distal colon but not in ileum explants, mucus grows at a steady state rate of ~ 2 µm/min. As mucus is continuously released from the goblet cells, and the mucus expands in volume following exocytosis, the parameter mucus growth over time is composed of two factors, baseline release rate and expansion of the secreted mucus [19]. To be able to determine whether certain anion transporters affected mucus expansion but not mucus release from the goblet cells, we first measured mucus growth over time and then processed the tissue for immuno-histological analysis to evaluate the mucus content of the tissue. By comparing these two parameters, we can determine whether a change in mucus growth rate was accompanied by an increase or decrease in mucus content of the tissue, indicative of a decrease or increase in mucus release from the tissue. A change in mucus growth without a parallel change in the mucus content of the tissue is interpreted as a change in mucus expansion.

## Materials and methods

### Mouse tissue

The experiments were performed using male and female homozygous CftrΔF508 (CF) and wild-type littermate controls on a C57/BL6 background [47]. Mice were housed in a specific pathogen-free facility, fed a routine chow diet, had access to water and food ad libitum, and kept at a 12 h light/dark cycle at a constant temperature (21–22 °C). Animals were between 8 and 15 weeks old at the time of the experiments. To increase the survival of the homozygous CftrΔF508 mice, they were given an osmotic laxative in the drinking water (PEG4000 18 mM, KCl 10 mM, Na_2_SO_4_ 40 mM, NaHCO_3_ 84 mM, and NaCl 25 mM). The animals were transferred to normal tap water 3 days prior to the experiments.

### Mucus thickness measurements

Measurements of mucus thickness were performed as described previously using apical Krebs mannitol and basal Krebs glucose buffer [19]. Briefly, the distal colon or ileum was dissected, flushed with ice-cold oxygenated Krebs buffer, and kept on ice for 30 min. Following incubation, the tissue was flushed once more with Krebs buffer, opened along the mesenteric border and the longitudinal muscle layer was removed by blunt dissection. The distal colon or ileum was divided into two parts and studied in parallel. To visualize the mucus layer, a suspension of activated charcoal particles was added to the apical surface and allowed to sediment down to the top of the mucus layer. The thickness of the distal colon mucus layer was assessed by measuring the distance between the mucus surface and the epithelial surface using a micropipette connected to a micromanipulator and a digimatic indicator. A schematic drawing of the method used to measure mucus is depicted in Gustafsson et al. [19]. In the ileum experiments, the tissue was mounted in the chamber, followed by the removal of the mucus by aspiration or scraping. The thickness of the remaining mucus was then measured by measuring the distance between the crypt openings and the mucus surface. Thus, in the ileum, the mucus thickness at t0 represents the mucus that is left in between the villi following removal. Carbachol (CCh) (1 mM), Prostaglandin E_2_ (PGE_2_) (10 µM), and the combination of CCh and PGE_2_ were added to the basal buffer, and the effect on mucus thickness was evaluated after 15/30 min in the colon and 20/40 min in the ileum. In the inhibitory experiments, DIDS (apical or basal), SLC26A3-IN-2, and the apical chloride-free buffer were added 30 min prior to CCh and PGE_2_ stimulation in the colon and 20 min prior to CCh and PGE_2_ stimulation in the ileum.

### Lectin staining

Distal colon tissues from the mucus measurement experiments were fixed in the measurement chamber for 1 h using 4% buffered formaldehyde (Histolab, Askim, Sweden), transferred to a tube containing fresh fixative and incubated overnight, followed by paraffin embedding and sectioning. Four micrometer thick sections were deparaffinized, rehydrated, and stained with fluorescein-conjugated UEA1 (25 µg/ml) (cat. no: FL-1061, Vector Laboratories, Burlingame, CA) and AlexaFluor 647 conjugated WGA (5 µg/ml) (cat no: W32466, Thermo Fisher Scientific, Waltham, MA) for 30 min followed, by 5 min wash in PBS and counterstained with Hoechst DNA stain for 5 min and imaged using a Nikon microscope. Acquired images were analyzed using Fiji [37] and Imaris (Bitplane, Belfast, Great Britain) software. A combination of UEA1 and WGA was used to stain the entire goblet cell population as UEA1 stains the surface goblet cells and goblet cells in the upper half of the crypt, while WGA stains goblet cells in the lower half of the crypt. WGA also stains the glycocalyx.

### Quantification of mucus secretion

Mucus secretion in the surface and crypt epithelium was quantified using Fiji [37] by measuring the sum of the theca areas of the surface or crypt goblet cells, divided by the area of either the surface or crypt area. To quantify the mucus content of the surface epithelium, the sum of the theca areas of all UEA1^+^ inter-crypt goblet cells was measured and divided by the area of the surface epithelium. To quantify the mucus content of the colonic crypts, the sum of the theca areas of all UEA1^+^ and/or WGA^+^ crypt goblet cells was divided by the area of the crypt cross section. Both the surface and crypt mucus content are expressed as percentage. Mucus secretion was defined as a significant decrease in mucus content of the respective compartment.

### Drugs and buffer composition

Carbamylcholine chloride (CCh) (cat no: C4382, P0409, Sigma-Aldrich, Steinheim, Germany) was dissolved in water. Prostaglandin E_2_ (PGE_2_) was dissolved in 50:50 ethanol and DMSO. 4,4′-diisothiocyanatostilbene-2,2′-disulfonate (DIDS) (cat no: S347523, Sigma-Aldrich, Steinheim, Germany) and SLC26A3-IN-2 (MedChemExpress, Sollentuna, Sweden) were dissolved in DMSO. Krebs buffer had the following composition in mM: NaCl 115.8, CaCl_2_ 1.3, KCl 3.6, KH_2_PO_4_ 1.4, NaHCO_3_ 23.1, and MgSO_4_ 1.2 (Merck, Darmstadt, Germany). The Krebs-mannitol buffer also contained Na-Pyruvate (5.7 mM) (Sigma-Aldrich, Steinheim, Germany), Na-L-Glutamate (5.1 mM) (Merck, Darmstadt, Germany) and D-Mannitol (10 mM) (Sigma-Aldrich, Steinheim, Germany), and the Krebs-glucose buffer contained Na-Pyruvate (5.7 mM), Na-L-Glutamate (5.1 mM), and D-Glucose (10 mM) (Sigma-Aldrich, Steinheim, Germany). In the chloride-free experiments NaCl, KCl, and CaCl_2_ were replaced with equimolar concentrations of Na-gluconate and K-gluconate, and twice the concentration of Ca-gluconate (Sigma-Aldrich, Steinheim, Germany) to compensate for the chelation of calcium by gluconate. pH was set to 7.4 using acetic acid.

### Statistics

Data are presented as mean ± standard error of the mean (SEM). Single comparisons between two groups were made using the Mann–Whitney test. Comparisons between two groups over time were made using a two-way ANOVA with Sidaks’ post-hoc test. Comparisons between three groups or more were made using the Kruskal–Wallis test with Dunns’ post-hoc test. A *p*-value < 0.05 was considered statistically significant.

## Results

### Steady-state mucus expansion in the distal colon is mediated by the apical anion exchanger slc26a3

**Fig. 1:**
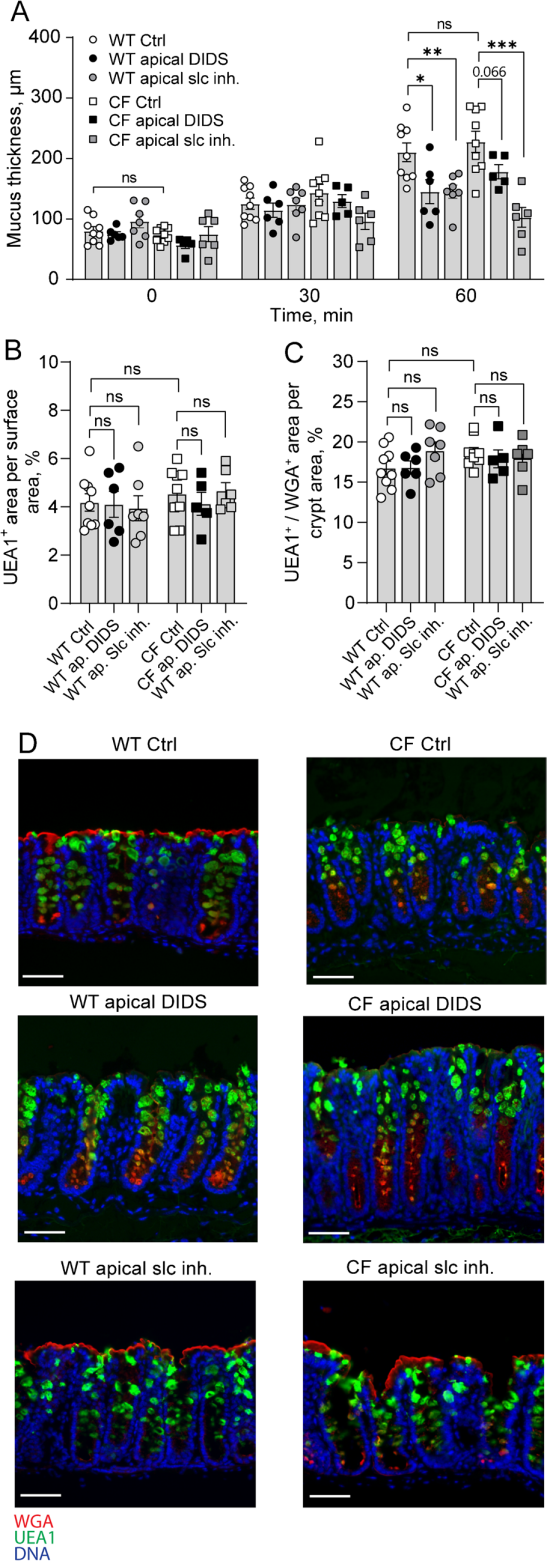
Steady state mucus expansion in the distal colon is mediated by the apical anion exchanger slc26a3. **A** Mucus thickness over time in WT ctrl (*n* = 9), WT treated with apical DIDS (200 µM) (*n* = 6) at t0, WT treated with apical SLC26A3-IN-2 (slc inh.) (*n* = 7), CF ctrl (*n* = 9) and CF treated with apical DIDS at t0 (*n* = 5), and CF treated with apical SLC26A3-IN-2 (slc inh.) (*n* = 6). **B** UEA1^+^ mucus area per epithelial surface area. **C** UEA1^+^/WGA.^+^ mucus area per distal colon crypt area. **D** Representative wide-field fluorescent images of WT and CF distal colon. Scale bar: 50 µm. Data are presented as mean ± SEM, *p < 0.05, **p < 0.01, ***p < 0.001, ns = non-significant. Statistical analysis was performed using a two-way ANOVA with Sidaks post-hoc test (**A**) and Kruskal–Wallis test with Dunn’s post-hoc test (**B**, **C**)

Previous studies have shown that in the small intestine, loss of Cftr-mediated bicarbonate secretion renders the normally loose and permeable mucus into a dense adherent structure [18]. To explore the role of Cftr in the regulation of mucus formation in the colon, we measured baseline mucus thickness and growth over time in the distal colon of C57/BL6 (WT) and CftrΔ508 (CF) mice and evaluated the mucus content of the tissues by lectin staining. Our results showed no difference in either the initial mucus thickness or baseline mucus growth comparing WT and CF distal colon (Fig. 1A). Analysis of the mucus content of the tissue showed no difference between WT and CF mice in either the surface epithelium (Fig. 1B, representative pictures in D) or crypt epithelium (Fig. 1C, representative pictures in D).

In addition to the Cftr, the distal colon expresses calcium-activated anion transporters of the anoctamin family and, anion exchangers that take up Cl^−^ and release bicarbonate into the colonic lumen of which slc26a3 (DRA) is highly expressed in the surface epithelium [22]. To explore whether other anion transporters than the Cftr are involved in regulating mucus formation in the colon we repeated the experiments in the presence of the broad-spectrum anion transport inhibitor 4,4′-diisothiocyanatostilbene-2,2′-disulfonate (DIDS), and the Slc26a3 inhibitor SLC26A3-IN-2. The results showed that both DIDS (200 µM), and SLC26A3-IN-2 (10 µM) reduced baseline mucus growth in the WT colon (Fig. 1A). In the CF colon, apical DIDS showed a trend towards reducing baseline mucus growth, while SLC26A3-IN-2 reduced baseline mucus growth by 80% (Fig. 1A). Analysis of the mucus content of the tissue showed no effect of either DIDS or SLC26A3-IN-2 in either the surface or crypt epithelium, respectively (Fig. 1B, C, representative pictures in D). Together these results demonstrate that steady-state mucus formation in the distal colon is independent of a functional Cftr channel but dependent on Slc26a3-mediated anion exchange.

### Effect of PGE_2_ and CCh in regulation of mucus layer formation in WT and CF distal colon

**Fig. 2:**
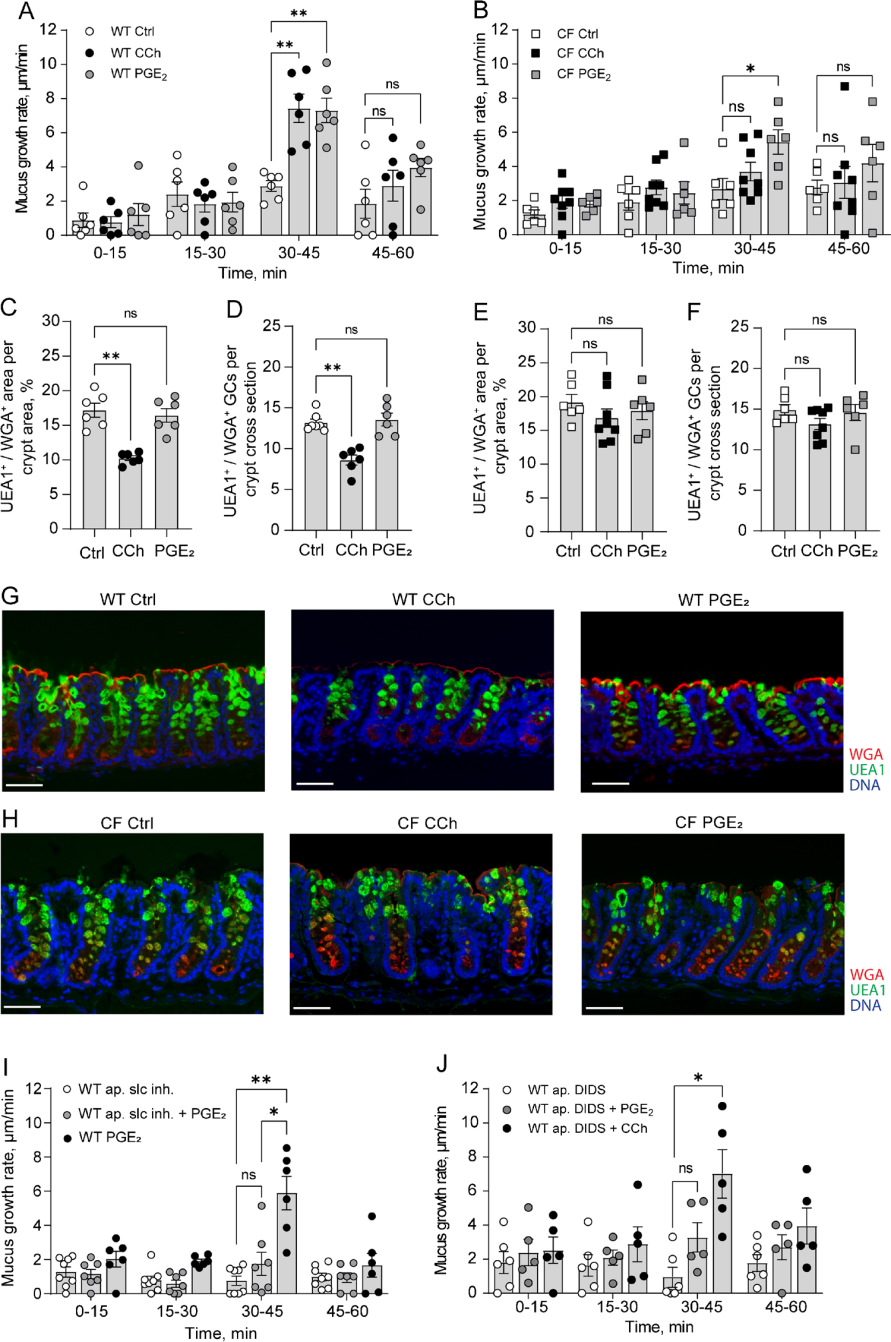
Effect of PGE_2_ and CCh in the regulation of mucus layer formation in WT and CF distal colon. **A** Mucus growth rate in WT ctrl (*n* = 6), WT treated with CCh (1 mM) (*n* = 6) at t30, WT treated with PGE_2_ (10 µM) (*n* = 6) at t30. **B** Mucus growth rate in CF ctrl (*n* = 6), CF treated with CCh (1 mM) (*n* = 8) at t30, CF treated with PGE_2_ (10 µM) (*n* = 6) at t30. **C** UEA1^+^/WGA^+^ mucus area per WT distal colon crypt area. **D** Number of UEA1^+^/WGA^+^ goblet cells per WT distal colon crypt cross section. **E** UEA1^+^/WGA^+^ distal colon mucus area per CF distal colon crypt area. **F** Number of UEA1^+^/WGA.^+^ goblet cells per CF distal colon crypt cross section. **G** Representative wide-field fluorescent images of WT distal colon. **H** Representative wide-field fluorescent images of CF distal colon. **I** Mucus growth rate in WT distal colon treated with apical SLC26A3-IN-2 at t0 (10 µM) (*n* = 8), WT treated with SLC26A3-IN-2 (slc inh.) (10 µM) + PGE_2_ (10 µM) (*n* = 7) and WT treated with PGE_2_ (*n* = 6). **J** Mucus growth rate in WT distal colon treated with apical DIDS at t0 (200 µM) (*n* = 5), WT treated with apical DIDS at t0 and PGE_2_ (10 µM) at t30 (*n* = 5), and WT treated with apical DIDS at t0 and CCh (1 mM) at t30 (*n* = 5). Scale bar, 50 µm. Data are presented as mean ± SEM, *p < 0.05, **p < 0.01, n.s = non-significant. Statistical analysis was performed using a two-way ANOVA with Sidaks’ post-hoc test (**A**, **B**, **I**, and **J**) and Kruskal–Wallis test with Dunn’s post-hoc test (**C**, **D**, **E**, and **F**)

To further characterize the role of anion transport in the regulation of mucus formation in the distal colon, we exposed WT and CF tissues to two secretagogues, the acetylcholine agonist CCh and PGE_2_. We assessed the effect of the two substances on mucus layer formation by measuring changes in mucus growth rate, mucus content of the tissue and the number of mucus-containing goblet cells. In WT mouse distal colon, exposure to CCh triggered a transient increase in mucus growth rate, induced a reduction in the mucus content of the crypt epithelium, and reduced the number of mucus-containing crypt goblet cells, indicative of complete emptying of a subset of goblet cells (Fig. 2A, C, D, representative pictures in G). Stimulation of WT distal colon tissues with PGE_2_ induced a transient increase in mucus growth rate, similar to that observed in response to CCh (Fig. 2A). However, PGE_2_ did not affect the mucus content of the tissue or the number of mucus-containing goblet cells (Fig. 2C, D, representative picture in G), suggesting that PGE_2_ regulates expansion of previously secreted mucus rather than stimulating de novo mucus secretion.

In CF mouse distal colon, exposure to CCh failed to induce an increase in mucus growth rate (Fig. 2B), and no effect was observed with respect to the mucus content of the tissue, or the number of mucus-containing goblet cells (Fig. 2E, F, representative pictures in H). In contrast, PGE_2_ induced a transient increase in the mucus growth rate, similar to that observed in WT distal colon (CF: 5.43 ± 0.8 vs WT: 7.3 ± 1.4, *p* > 0.05) (Fig. 2B). PGE_2_ did not affect the mucus content of the tissue or the number of mucus containing goblet cells in CF distal colon, similar to that observed in WT distal colon (Fig. 2E, F, representative pictures in H).

Based on our results demonstrating that baseline mucus growth was regulated by slc26a3, we proceeded to explore whether the PGE_2_-induced effect on mucus growth was regulated by similar mechanisms. Our results showed that pretreatment of the WT distal colon with apical SLC26A3-IN-2 inhibited the PGE_2_ effect on mucus growth rate (Fig. 2I). A similar effect was observed with apical DIDS that inhibited the PGE_2_ response but had no effect on CCh induced mucus growth (Fig. 2J). Combined, these results suggest that in the distal colon, CCh induces de novo mucus secretion via Cftr-dependent processes, while PGE_2_ induces mucus expansion via Slc26a3-dependent processes.

### Stimulation of CF distal colon with the combination of CCh and PGE_2_ induces de novo mucus secretion

**Fig. 3:**
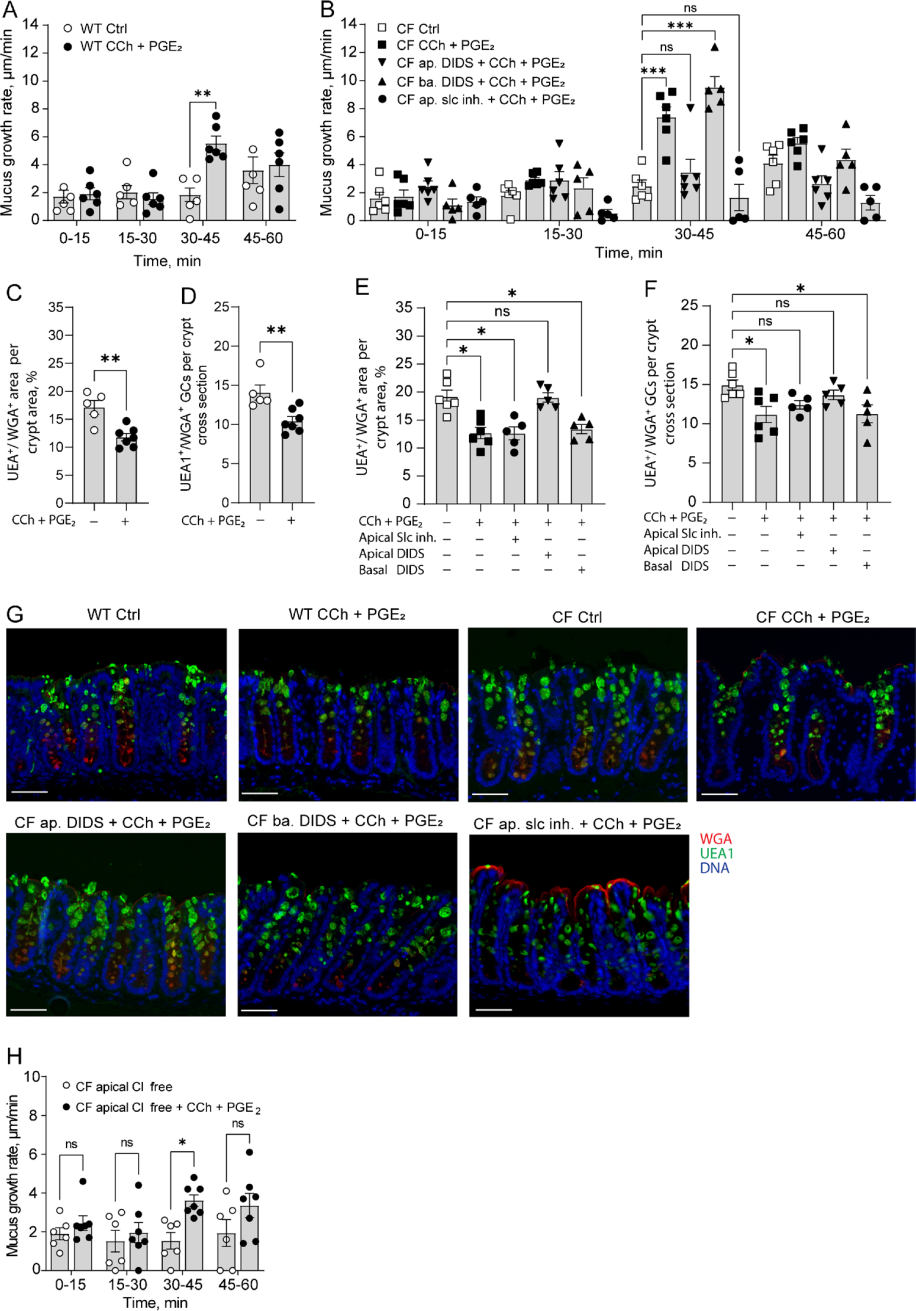
Parallel stimulation of CF distal colon with CCh and PGE_2_ induces de novo mucus secretion. **A** Mucus growth rate in WT ctrl (*n* = 5) and WT treated with CCh (1 mM) and PGE_2_ (10 µM) at t30 (*n* = 6). **B** Mucus growth rate in CF ctrl (*n* = 6), CF treated with CCh (1 mM) and PGE_2_ (10 µM) at t30 (*n* = 6), CF treated with apical DIDS (200 µM) at t0 followed by CCh (1 mM) and PGE_2_ (10 µM) at t30 (*n* = 6), CF treated with basal DIDS (200 µM) at t0 followed by CCh (1 mM) and PGE_2_ (10 µM) at t30 (*n* = 5), and CF treated with apical SLC26A3-IN-2 (slc inh.) (10 µM) at t0 followed by CCh (1 mM) and PGE_2_ (10 µM) at t30 (*n* = 6). **C** UEA1^+^/WGA^+^ distal colon mucus area per WT crypt cross section. **D** Number of UEA1^+^/WGA^+^ goblet cells per WT distal colon crypt cross section. **E** UEA1^+^/WGA^+^ distal colon mucus area per CF crypt cross section. **F** Number of UEA1^+^/WGA^+^ goblet cells per CF distal colon crypt cross section. **G** Representative wide-field fluorescent images of WT and CF distal colon. **H** Mucus growth rate in CF treated with apical Cl^−^ free buffer at t0 (*n* = 6), and CF treated with apical Cl^−^ free buffer at t0 and CCh (1 mM) and PGE_2_ (10 µM) at t30 (*n* = 7). Scale bar, 50 µm. Data are presented as mean ± SEM, *p < 0.05, **p < 0.01, ***p < 0.001, n.s = non-significant. Statistical analysis was performed using a two-way ANOVA (**A**, **B**, and **H**) with Sidaks’ post-hoc test, non-paired Mann-Whitney test (**C**-**D**) and Kruskal–Wallis test with Dunn’s post-hoc test (**E**–**F**)

Based on our finding of an impaired mucus secretory response to CCh but an intact mucus growth response to PGE_2_ in CF distal colon, and previous studies demonstrating that parallel activation of Ca^2+^ and cAMP-mediated pathways can help release mucus from GCs [49], we explored whether activation of PGE_2_ mediated transport pathways could normalize the impaired CCh response in CF distal colon. Distal colon tissues from WT and CF mice were exposed to a combination of CCh (1 mM) and PGE_2_ (10 µM), and the effect on mucus growth rate, mucus content in the tissue, and the number of mucus-containing goblet cells were measured. Stimulation of tissues with CCh and PGE_2_ induced a transient increase in mucus growth rate in both WT (Fig. 3A) and CF distal colon (Fig. 3B) that was paralleled by a decrease in the mucus content of the tissue and a decrease in the number of mucus containing goblet cells in both WT (Fig. 3C, D, representative pictures in G) and CF (Fig. 3E, F, representative pictures in G) distal colon. To determine whether the de novo mucus secretory response observed in the CF distal colon involved activation of slc26a3, the experiments were repeated in the presence of SLC26A3-IN-2. Pretreatment with SLC26A3-IN-2 for 30 min inhibited the CCh + PGE_2_-induced increase in mucus growth rate, and reduction in mucus-containing cells (Fig. 3B, F, representative image in G), but did not inhibit the CCh + PGE_2_ induced reduction in the mucus content of the tissue (Fig. 3E, representative image in G). To determine whether additional anion transporters were activated in response to CCh + PGE_2_, the experiments were repeated in the presence of apical or basolateral DIDS. Pretreatment with apical DIDS inhibited the CCh + PGE_2_-induced increase mucus growth rate, the reduction in mucus content in the tissue, and the number of mucus-containing goblet cells (Fig. 3B, E, F, representative pictures in G). In contrast, 30 min pretreatment with DIDS added to the basal side of the tissue had no effect on either of the three parameters (Fig. 3B, E, F, representative pictures in G). To validate the involvement of apical anion exchange in regulating the CCh + PGE_2_-induced increase in mucus growth rate, we repeated the experiments using chloride-free apical buffer to inhibit Cl^−^/HCO_3_^−^ exchange. Removal of chloride from the apical buffer reduced the CCh + PGE_2_ response by 90% (Fig. 3H), further supporting a role for Cl^−^/HCO_3_^−^ exchange in regulating secretagogue-induced mucus expansion. Combined, these results suggest that the combination of CCh and PGE_2_ activates multiple transport processes that trigger mucus release from goblet cells and expansion of the secreted mucus even in the absence of a functional Cftr channel.

### Effect of CCh and PGE_2_ in regulating mucus layer formation in the WT and CF ileum

**Fig. 4:**
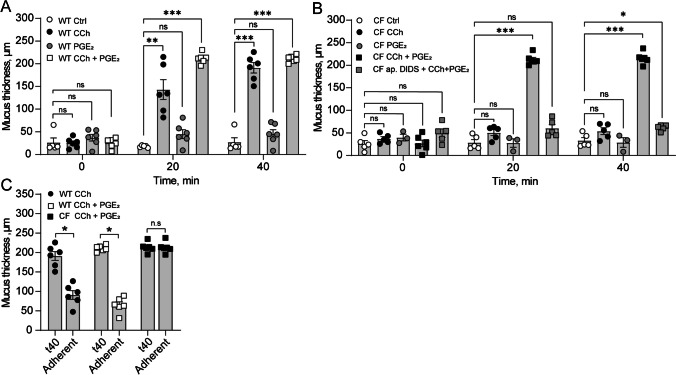
Effect of CCh and PGE_2_ in regulating mucus layer formation in the WT and CF ileum*.* **A** Mucus thickness in WT Ctrl (*n* = 5), WT treated with CCh (10 µM) (*n* = 6), WT treated with PGE_2_ (10 µM) (*n* = 6), and WT treated with CCh and PGE_2_ (10 µM + 10 µM) (*n* = 6). **B** Mucus thickness in CF Ctrl (*n* = 5), CF treated with CCh (10 µM) (*n* = 5), CF treated with PGE_2_ (10 µM) (*n* = 3), CF treated with CCh and PGE_2_ (10 µM + 10 µM) (*n* = 6), and CF treated with apical DIDS (200 µM) + CCh (10 µM) + PGE_2_ (10 µM) (*n* = 5). **C** Mucus adhesion in WT CCh, WT CCh + PGE_2_, and CF CCh + PGE_2_. CCh, PGE_2_, and the combination of CCh + PGE_2_ were added directly after the first thickness measurement (t0). DIDS was added 20 min prior to the first thickness measurement. Data are presented as mean ± SEM, *p < 0.05, **p < 0.01, ***p < 0.001, n.s = non-significant. Statistical analysis was performed using a two-way ANOVA with Sidak’s post-hoc test (**A** and **B**) and a paired Mann–Whitney test (**C**)

In the small intestine, loss of Cftr-mediated transport results in mucus adhering to the tissue [18]. We have previously shown that stimulation of ileal tissues with the combination of CCh and PGE_2_ induces de novo mucus secretion in both WT and CF tissues, with the main differences between the groups being that the secreted mucus is more dense and adherent to the tissue [18]. Based on our findings from the distal colon, we asked whether CCh induced de novo mucus secretion is impaired also in the CF ileum, and whether the previously observed intact mucus secretory response to CCh and PGE_2_ relies on the activation of additional apical anion transporters. To address these questions, we exposed WT and CF ileal explants to CCh (10 µM), PGE_2_ (10 µM), and the combination of CCh (10 µM) and PGE_2_ (10 µM) and measured the effect on mucus thickness over time and mucus adhesion. In the WT ileum, stimulation with CCh induced an increase in mucus thickness, while PGE_2_ had no effect on the mucus thickness (Fig. 4A). Stimulation of WT ileal tissues with the combination of CCh and PGE_2_ induced an increase in mucus thickness similar in magnitude to that observed in response to CCh (Fig. 4A). In the CF ileum, neither CCh nor PGE_2_ induced an increase in mucus thickness when added separately (Fig. 4B). However, stimulation of CF ileum with the combination of CCh and PGE_2_ induced a significant increase in mucus thickness, similar in magnitude to that observed in WT ileum (Fig. 4B). To test whether the CCh and PGE_2_ induced increase in mucus thickness in CF ileum could be blocked by inhibition of anion transport, we repeated the experiments in the presence of apical DIDS. Pretreatment with apical DIDS reduced the CCh and PGE_2_-induced increase in mucus growth in CF ileum by 84.2% (Fig. 4B). To test the adhesive properties of the secreted mucus, we aspirated the mucus and measured the remaining thickness. In the WT ileum, 57 ± 5.0% of the CCh-induced mucus, and 78 ± 3.8% of the CCh + PGE_2_-induced mucus could be aspirated, while in the CF ileum, all of the CCh + PGE_2_-induced mucus remained attached to the tissue (Fig. 4C). Combined, these results suggest that in the ileum, CCh induces mucus secretion via Cftr-mediated pathways and that in the absence of a functional Cftr channel, PGE_2_ can help release mucus from the goblet cell by activation additional apical transport pathways.

## Discussion

In both the small and large intestines, maintaining a functional mucus barrier is essential to maintain homeostasis. In the small intestine, a loose and permeable mucus barrier allows for efficient nutrient absorption and facilitates the transport of waste products and microorganism to the colon. In the mid to distal colon, the mucus layer transforms into a physical barrier separating the vast majority of live microbes from accessing the underlying epithelium [5, 27]. How the intestines regulate mucus properties to fit the needs of the respective organs is not fully understood but the ionic milieu in the intestinal lumen is known to play an important role in regulating mucus formation by affecting mucin granule exocytosis, mucin expansion, and mucus adhesion to the epithelium [12, 18, 20]. In this study, we discovered that the apical anion exchanger slc26a3 regulates mucus expansion in the distal colon both during steady state and in response to the secretagogue PGE_2_. Cftr, on the other hand, was shown to regulate CCh-induced de novo mucus secretion in both the small intestine and distal colon and to regulate mucus adhesion in the small intestine.

In the intestines, anions can be transported into the lumen via several transport processes including the anion channels, Cftr and Tmem16A (Anoctamin 1), and a number of anion exchangers of which the Cl^−^/HCO_3_^−^ exchangers Slc26a3 and Sc26a6 that are expressed in the colon and ileum, respectively are the most characterized [9, 31, 46, 48]. As mentioned previously, studies have shown that Cftr plays an essential role in regulating mucus formation in the small intestine by preventing mucus adhesion to the tissue, and in the context of mucus, it is the transport of bicarbonate into the intestinal lumen that is particularly important to ensure that the mucus is released and expanded correctly [1, 8, 18, 35, 49]. In the colon, the Ca^2+^-regulated anion channel Tmem16A (Anoctamin 1) has been implicated in the regulation of baseline mucus release, and Tmem16A expression has been shown to be upregulated in response to Th2 cytokines that also drives mucus secretion [4, 40]. However, the knowledge regarding the role of additional transporters in the regulation of mucus expansion following secretion, and how anion transport is involved in regulating induced secretion is lacking. In the present study, we demonstrate that baseline mucus growth in the colon is stimulated by the anion exchanger slc26a3, and independent of a functional Cftr channel. Since inhibition of slc26a3 did not affect the mucus content of the tissue, we interpret this to mean that slc26a3 drives the expansion of previously secreted mucus rather than stimulating de novo mucus release from goblet cells. Taken together, these findings demonstrate that both Tmem16A (Anoctamin 1) and Slc26a3 are involved in regulating steady-state mucus formation in the colon by stimulating mucus release and expansion of the secreted mucus. However, although the slc26a3 inhibitor and DIDS reduced baseline mucus growth in the WT distal colon, mucus growth was not completely inhibited, pointing towards additional mechanisms being involved in regulating colonic mucus expansion following secretion. Proteolytic processing of the Muc2 has been shown to occur in both the small intestine and colon, which may account for the residual mucus growth observed in the presence of the anion exchange inhibitor SLC26A3-IN-2 and DIDS [34, 39]. Due to the fact that the ileum does not exhibit a measurable steady state mucus growth ex vivo [13, 18], we were not able to evaluate the role of anion exchange in the regulation of baseline mucus expansion in the ileum.

In both the small and large intestine, mucus is secreted at a steady state rate but can also be induced by Ca^2+^ mobilizing agents such as acetylcholine and its analogue CCh resulting in compound exocytosis [6, 12, 19, 42]. In both the small and large intestines, exposure to CCh induces a rapid increase in intracellular Ca^2+^ levels which triggers mucin granule exocytosis and activates transcellular anion transport and subsequent fluid secretion that helps expanding the released mucus [42]. Although the Cftr is not directly regulated by Ca^2+^, activation of Ca^2+^ dependent K^+^ channels will trigger an influx of anions ions that leave the cell via the Cftr, thus a large part of Ca^2+^ mediated anion secretion is mediated by the Cftr [14, 20, 38]. Previous studies have shown that CCh-induced mucin granule fusion with the plasma membrane remains intact in CF distal colon tissues, while studies in small intestine organoids from CF mice show that mucus remains within the goblet cells following CCh stimulation, and that stimulation of CF organoids in the presence of high concentrations of bicarbonate can restore the impaired mucus secretory response [12, 20, 28]. Our results confirm that the same pattern with mucus remaining within the goblet cells following CCh stimulation is observed in intact tissue specimens from CF distal colon, which supports a role for Cftr in driving CCh-induced mucus release from goblet cell in vivo.

In addition to testing the role of anion transport in the regulation of CCh-induced mucus secretion, we evaluated the role of PGE_2_ in regulating mucus layer formation. PGE_2_ is a well-established regulator of mucus and bicarbonate secretion in the stomach where it protects the stomach epithelium from the acidic luminal content [44, 45]. In the intestine, the role of PGE_2_ in regulating mucus secretion appears to be species dependent. In rat colon, PGE_2_ induces de novo mucus secretion and increases mucus output [2]. In contrast, studies in human colonic explants and mouse ileum have not observed de novo mucus secretion in response to PGE_2_, but PGE_2_ has been shown to induce mucus expansion in human colonic primary cultures [21, 41, 49]. In the present study, we show that in the mouse distal colon, stimulation with PGE_2_ induces an increase in mucus growth rate without signs of de novo mucus release. We interpret this to mean that PGE_2_ induces the expansion of already released mucus. Our results showed that the PGE_2_-induced increase in mucus growth was dependent on slc26a3 activity. Studies in mouse small intestine have shown that PGE_2_-induced bicarbonate secretion is mediated by slc26a6, and a similar process may be present in the colon mediated by slc26a3 [46]. In the mouse ileum experiments, PGE_2_ did not stimulate an increase in mucus thickness, which correlates with previous findings demonstrating that PGE_2_ does not induce de novo mucus release in the small intestine. The observed species differences in the effect of PGE_2_ in regulating de novo mucus secretion may be related to species differences in prostaglandin receptor (Ptger) expression. Mouse colonic goblet cells primarily express Ptger4 that signals via cAMP, and to a lower extent Ptger1 that signals via Ca^2+^, and Ptger3 that inhibits cAMP production [43]. A similar pattern is seen in human colonic goblet cells that express low levels of Ptger4, 1, and 3 [26]. Rat colonic goblet cells, on the other hand, express all four Ptgers, and thus have the possibility to induce stronger Ca^2+^ responses triggering de novo mucus secretion [33].

Despite our findings that neither CCh nor PGE_2_ triggered de novo mucus secretion in CF ileum and colon when added separately, our results demonstrate that when added simultaneously, they trigger de novo mucus secretion in both organs. Our finding that the mucus growth rate but not the mucus content of the tissues was reduced by slc26a3 inhibition, suggests that slc26a3 drives mucus expansion, but that additional DIDS-sensitive transporters are activated in response to the combination of CCh and PGE_2_ that drive mucus release from the goblet cells. The identity of the DIDS-sensitive transporter(s) involved in this process is not known but may involve Tmem16A (Anoctamin 1), as mice lacking Tmem16A in intestinal epithelial cells have been shown to have impaired secretory responses to both Ca^2+^ and cAMP mediated agonist [3]. However, despite responding with de novo mucus secretion, in the CF small intestine, the combination of CCh and PGE_2_ did not reverse mucus adhesion to the tissue. This observation, that activation of parallel secretory pathways (Ca^2+^ and cAMP), results in a normal mucus secretory response in the colon, but secretion of adherent mucus in the small intestine may explain the more severe mucus pathology in the small intestine as compared to the colon. In vivo, Ca^2+^ and cAMP mediated pathways will be active in parallel by substances such as Ach, VIP, histamine, 5-HT, and PGE_2_, that all signal via G protein coupled receptors. In the small intestine, this will result in release of adherent mucus that triggers a vicious circle of mucus secretion, obstructions, and inflammation. In the colon, on the other hand, as Cftr is not involved in regulation of baseline mucus formation, and that parallel activation of Ca^2+^ and cAMP-mediated pathways restore Ca^2+^ mediated mucus release from the colonic crypts, may allow for the formation of a mucus layer that protects the epithelium from the microbiota, thus explaining the less severe pathology in CF colon [17].

In conclusion, this study demonstrates that anion transport via the Cftr and the apical anion exchanger Slc26a3 are involved in regulating mucus layer formation during steady state and in response to secretagogues. Apical anion exchange via Slc26a3 appears to induce expansion of already secreted mucus in the distal colon, while Cftr drives de novo mucus secretion in response to cholinergic stimulation.

## Data Availability

All mentioned data are represented in the main manuscript figures. Other additional data will be made available on reasonable request.
